# Efficacy and safety of oral traditional Chinese patent medicine for chronic cerebral circulation insufficiency patients: A protocol for a systematic review and network meta-analysis

**DOI:** 10.1097/MD.0000000000016175

**Published:** 2019-07-05

**Authors:** Zhongbo Xu, Xin Feng, Lin Li, Ziyi Hu, Yuan Xiao, Guilin Jin, Weimin Liao

**Affiliations:** a Affiliated Hospital of Jiangxi University of Traditional Chinese Medicine; b Maternal and Child Health Hospital of Jiangxi Province, China.

**Keywords:** chronic cerebral circulation insufficiency, network meta-analysis., oral traditional Chinese patent medicine, protocol, systematic review

## Abstract

**Background:**

Chronic cerebral circulation insufficiency (CCCI) is a common clinical cerebrovascular disease, especially among middle-aged and elderly patients, which seriously endangers their quality of life and physical and mental health. At present, Oral traditional Chinese patent medicine (OTCPM) is widely used in the treatment of CCCI in China, but its actual efficacy and safety lack of evidence-based evidence. Therefore, we will screen out the most effective OTCPM through a systematic review and network meta-analysis to provide a reliable theoretical basis for clinical decisions.

**Methods:**

We will search electronic databases to collect relevant RCT studies from inception to October 2019. Those electronic databases include PubMed, Cochrane Library, Web of Science, EMBASE, China Biomedical Literature Database (CBM), China National Knowledge Infrastructure (CNKI), Chinese Scientific Journal Database (VIP), and Wan-fang database. Only randomized clinical trials (RCTs) concerned any OTCPM treatments for CCCI will be collected. The included studies will no restrictions on language or publication year. There were no publication year or language for the included literature. Risk bias tools will assess the quality of the included literature. A Bayesian NMA will be performed to combine the direct and indirect comparisons of TCPMs interventions. The surface under the cumulative ranking curve (SUCRA) will be drawn to display the hierarchy of each TCPMs treatment. All statistical analyses will be implemented using R v3.5.2. and GeMTC v1.4.3.

We will publish this systematic review in academic journals. Since this literature review will not involve directly contacting patients, ethical approval and informed consent are not required.

**Trial registration number:**

CRD42019123878.

## 1 Introduction

Chronic cerebral circulation insufficiency (CCCI) is a cerebral arterial circulation disorder resulted from decreased cerebral blood flow, which can cause dizziness, dizziness, headache, insomnia, forgetfulness, and other manifestations of cerebrovascular diseases.^[1,2]^ CCCI is considered to be associated with the development of diseases such as ischemic stroke, Alzheimer disease, vascular dementia, and other cerebrovascular diseases. ^[3,4]^ According to statistics, about two-thirds of the elderly than 65 years of age will have different levels of chronic cerebral insufficiency in China. ^[2]^ Despite the high incidence of CCCI, modern medicine currently lacks specific treatment. The main treatment plan is to actively control the underlying diseases with lipid-lowering, antihypertensive and hypoglycemic drugs, and at the same time to give cerebral vasodilator drugs such as nitrendipine and flunarizine. In China, traditional Chinese medicine (TCM) has a great advantage in the treatment of CCCI, and patients are more inclined to choose TCM for the treatment of CCCI.

Traditional Chinese patent medicine refers to a kind of medicine made by a certain proportion of a variety of Chinese herbal medicines, including oral dosage forms such as granules, capsules, tablets, and dripping pills, as well as those that can be injected intravenously. Oral traditional Chinese patent medicine (OTCPM) is a crucial part of TCM, which has been widely accepted and applied in clinical practice in China. Compared with Chinese herbal medicines, OTCPM are more convenient to take, easy to carry, no odor and irritating, and many patients with CCCI are willing to choose OTCPM. In this case, the studies about OTCPM for treating CCCI is also increasing. Recently, some scholars have carried out a conventional pairwise meta-analysis to evaluate the efficacy of certain OTCPM in the treatment of patients with CCCI, ^[5]^ which has certain guiding significance for clinical decision-making. However, with the increasing number of OTCPM, traditional meta-analysis has been unable to provide a basis for the selection of the best intervention drugs. Hence, we will use the Network Meta-Analysis (NMA) to conduct an evidence-based evaluation on the clinical efficacy and safety of OTCPM in the treatment of CCCI, and select the best intervention measures, so as to provide theoretical reference for clinical medication.

## 2 Methods and analysis

We will complete this System Review and NMA protocol, according to Protocol the preferred reporting requirements of the System Review and Meta-Analysis Protocol (PRISMA-P) statement. ^[6]^ The report of the further results of this study will be presented according to the guidelines of the PRISMA extension statement for network meta-analyses. ^[7]^ We have registered this protocol in the PROSPERO network on March 2019(CRD42019123878).

### 2.1 Inclusion criteria

#### 2.1.1 Types of studies

The literature only included randomized controlled trials (RCTs), which were not limited by blinding, language, and publication time.

#### 2.1.2 Types of patients

Patients with a diagnosis of CCCI will be included. The diagnostic criteria for CCCI will be developed according to the previous publication.^[8]^ The included:

1. dizziness and headache are the main symptoms, accompanied by sleep disorders (such as difficulty falling asleep, short sleep time, easy to wake up), memory loss and other clinical symptoms;
2. evidence of cerebral arteriosclerosis: fundus arteriosclerosis was changed, sometimes vascular noises of cerebral perfusion could be heard, and transcranial doppler (TCD) showed decreased cerebral blood flow velocity and increased pulse index;
3. no signs of focal nerve localization in the brain were found in the neurological examination;
4. no abnormalities or slight ischemic changes were found in CT or MRI of the head;
5. cervical vascular color doppler ultrasonography showed carotid endometrial thickness (IMT) ≥0.10 cm, or carotid atherosclerotic plaque or lumen stenosis;
6. aged above 45;
7. exclude cardiac, anemia, infectious diseases, cervical spondylosis, ear diseases, and other systemic diseases causing the above symptoms.

#### 2.1.3 Types of interventions

In the case of basic disease control and treatment, the intervention measures of the experimental group were OTCPM, whereas the control group were conventional drug therapy or placebo. The intervention measures in the 2 groups did not include intravenous, traditional Chinese medicine decoction or acupuncture, and other traditional Chinese treatment methods. Traditional Chinese patent medicine refers to a kind of medicine made by a certain proportion of a variety of Chinese herbal medicines, included different dosage forms including pills, capsules, granules, and tablets.

#### 2.1.4 Types of outcome measures

Clinical effectiveness indicators in eligible studies should be include: hemorheology; mean blood flow velocity in various cerebral arteries, internal carotid arteries, vertebral arteries, and basilar arteries; clinical evaluation of cognitive function: mainly using the Mini-mental State Examination (MMSE) and Hasegawa Dementia Scale (HDS) performs clinical evaluation of cognitive function. Safety outcome should be include rates of discontinuation and treatment-related adverse events between 2 groups.

### 2.2 Exclusion criteria

Exclusive criteria included:

1. No-RCT literature, case reports, reviews, animal experiments, conference papers, duplicate, or published literature;
2. the original literature design is not rigorous, and there is no statistical method and comparison;
3. a document that is incomplete and inaccessible.

#### 2.2.1 Search strategy

Relevant RCT studies will be searched in the following Chinese and English electronic databases from inception to October 2019 including CNKI, CBM, Chinese Scientific Journal Database, Cochrane Library, EMBASE, PubMed, Web of Science, and Wan-fang database. According to the “PICOS” search principle, the subject words used were as follows: “Chronic Cerebral Circulation Insufficiency”, “Chinese Patent Medicine”, “Capsule”, “Granule”, “Dropping Pills”, “RCT” and so on. The subject words and free words will be searched respectively, and related free words and entry words are used for the comprehensive search.

#### 2.2.2 Searching other resources

Meanwhile, we will search relevant journals and follow-up the relevant literature in the reference and use the search engines such as Google Scholar to find relevant documents on the Internet manually. We will ensure as comprehensive a search as possible so as not to miss relevant research.

### 2.3 Study selection

The literature will be independently selected and screened by 2 researchers according to the predetermined inclusion criteria and exclusion criteria. If there are have a disagreement between 2 researchers, they will be solved by the third researcher. The screening steps of the original literature are as follows: Firstly, duplicate studies will be removed by literature management software (EndNote X9); secondly, the researcher will browse the title and abstract of the literature to collect all possible relevant and certainly related researches; finally, the researchers will read the full text of the literature and determine the final included literature. The reasons for inclusion or exclusion of each article will be recorded in the process of screening original literature.

### 2.4 Data extraction and management

Data will be extracted from the selected literature and completed by 2 independent reviewers using a standardized extraction form. They will record the following study information: author, populations, published time, study design, intervention measures (dosage types of the 2 group, administration dose, frequency of administration, course of treatment) and outcomes indicators. Then, they will check the results for accuracy doubly. If there is a disagreement, we will resolve it by discussion or by a third researcher. For the resolution of the missing data in the literature, we will contact the corresponding author by phone or email to obtain the necessary data.

### 2.5 Assessment of risk of bias

Methodological quality of the included literature will be evaluated according to the criteria recommended by the Cochrane Handbook 5.3.1, which included

1. description of random allocation method and generation of sequence;
2. study the hiding of random allocation scheme;
3. the implementation of blinded subjects and researchers, the implementation of blind methods for outcome evaluators;
4. the integrity of report data;
5. the possibility of selectively reporting research results;
6. consider other sources of bias. Each item should be judged into a “yes” (low bias), “no” (high bias), and “unclear” (lack of relevant information or uncertainty about bias).

The 2 researchers will independently cross-check the quality evaluation results of the corresponding literature. If there is any disagreement in the results, they will discuss with each other or be determined by the third evaluator.

### 2.6 Statistical analysis

We will provide a descriptive summary of the qualified literature based on patient characteristics, study characteristics, interventions and outcome indicator, and risk of bias. The dichotomous data will be represented by calculating the odds ratios (OR) whereas continuous outcomes will be pooled with standardized mean difference (SMD). Each effect scale index will be calculated by 95% confidence intervals (95%CI).

Our systematic review will be implemented a two-step approach. Firstly, we will carry out a traditional pairwise meta-analysis so as to integrate all direct evidence. Secondly, we will synthesize both direct and indirect evidence by using an NMA. The Bayesian framework and the Markov Chain Monte Carlo (MCMC) sampling technique will be applied to generate summary means differences and summary ORs. Trace plots and density plots will be used to evaluate convergence of the simulations.^[9]^ Moreover, we will draw net relation diagram, contribution graph, inconsistency check chart, comparison-adjusted funnel plot, and forest plot. The cumulative ranking processed foe each OTCPM treatment is estimated by the surface under the cumulative ranking curve (SUCRA). A higher SUCRA value indicates better efficacy or safety, which is 1 when the treatment is determined as the best and 0 when the treatment is determined as the worst. ^[10]^

The statistics of Bayesian meta-analysis will be performed using GeMTC v1.4.3 software. Net relation diagram, contribution graph, inconsistency check chart, comparison-adjusted funnel plot, and forest plot will be drawn by R v3.5.2. The network meta-analysis will be undertaken in accordance with the PRISMA statement.^[11]^

#### 2.6.1 Assessment of heterogeneity

*We will use Chi-Square test and calculate I*^*2*^ index to assess heterogeneity. When *I*^*2*^*≥* 50%, we will consider that heterogeneity exists.^[12]^Under this condition, subgroup analysis or sensitivity analysis will be performed to investigate the source of heterogeneity. When the source of heterogeneity cannot be determined, we will implement the random-effect model. Otherwise, we will conduct the fixed-effect model.

#### 2.6.2 Assessment of inconsistency

The inconsistency between direct and indirect evidence will assess by using the node-splitting model, which can calculate the difference between direct and indirect evidence.^[13]^ We will determine if there is an inconsistency based on the *P* value.

#### 2.6.3 Assessment of similarity

The NMA similarity hypothesis can only be evaluated through an indirect method. Due to the limitations of qualitative evaluation, it is not guaranteed that all confounders will be found. Therefore, we will evaluate the similarity of NMA by comparing the similarity of clinical and methodological characteristics between studies.

## 3 Assessment of quality of evidence

We will apply the Grading of Recommendations Assessment, Development and Evaluation (GRADE) approach to analysis the overall quality level of evidence on the efficacy and safety of different OTCPM.^[14]^ The quality of RCT evidence will be classified, according to the following 5 factors:

1. limitations of design;
2. the indirectness of the evidence;
3. unknown heterogeneity of results;
4. inaccuracy of results;
5. probability of publication bias.

There are 4 levels high, medium, low or very low for the quality of RCT evidence.

## 4 Discussion

OTCPM has been widely used in treatment of patients with CCCI in clinical practice in China. To our knowledge, a lot of literature has been reported that OTCPM can effectively relieve dizziness, headache, insomnia, and other clinical symptoms in patients with CCCI.^[15]^ However, there is a lack of network meta-analysis on the effectiveness and safety of OTCPM in the treatment of CCCI. Therefore, we felt that we should compare the clinical efficacy of various oral Chinese patent medicines in the treatment of CCCI. Hence, we proposed this systematic review to evaluate efficacy and safety of OTCPM for CCCI, hoping this study will provide more convincing evidence to demonstrate the advantages of OTCPM for CCCI and provide a reference for clinical practice.

We anticipate that our research may have some potential limitations. First, potential heterogeneity could arise from the different doses of OTCPM and course of treatment. Second, there may have some small samples of trails, which may lead to high risks of bias. Third, inclusion of only Chinese and English studies may result in publication bias. After a comprehensive analysis, the above limitations will be described in detail in the discussion section of the review.

## Author contributions

**Conceptualization:** Zhongbo Xu, Xin Feng.

**Data curation:** Lin Li, Ziyi Hu.

**Formal analysis:** Yuan Xiao, Guilin Jin.

**Methodology:** Lin Li, Yuan Xiao, Guilin Jin.

**Software:** Xin Feng.

**Supervision:** Weimin Liao.

**Validation:** Weimin Liao.

**Writing – original draft:** Zhongbo Xu, Xin Feng.

**Writing – review & editing:** Zhongbo Xu.
